# Long-term effectiveness, safety, and tolerability of doravirine in antiretroviral-experienced people with HIV in real life

**DOI:** 10.1128/spectrum.00654-24

**Published:** 2024-06-25

**Authors:** Marta Mejías-Trueba, Alicia Gutierrez-Valencia, Silvia Llaves-Flores, Cristina Roca-Oporto, Marta Herrero, César Sotomayor de la Piedra, Luis F. Lopez-Cortes, Nuria Espinosa

**Affiliations:** 1 Department of Pharmacy, University Hospital Virgen del Rocío, Seville, Spain; 2 Infectious Diseases and Microbiology Clinical Unit. Instituto de Biomedicina de Sevilla/University Hospital Virgen del Rocío/CSIC/Universidad de Sevilla, Sevilla, Spain; 3 Primary Care Pharmacist Service, Sevilla Primary Care District, Sevilla, Spain; University of Miami, Miami, Florida, USA

**Keywords:** HIV cure, ARV, viral supression, treatment

## Abstract

Real-life data on doravirine (DOR) in different drug combinations are limited. We evaluated the effectiveness of DOR plus two nucleos(t)ide reverse transcriptase inhibitors (NRTI), mainly abacavir/lamivudine, and dual therapies in people with HIV (PWH), mostly virologically suppressed. Ambispective observational study that enrolled adults PWH who initiated a DOR-based regimen from September 2020 to February 2022 at a referral center in Spain. Participants were grouped as follows: A, received DOR plus two NRTI; B, dual therapy (DT) with DOR plus dolutegravir (DTG) or darunavir/cobicistat (DRVc); C, DOR plus ≥two antiretroviral drugs. The primary endpoints were treatment effectiveness at week 48 by intention-to-treat (ITT) and per-protocol analysis (OT). A cohort of 187 participants, 91% virologically suppressed, were analyzed after a median follow-up of 112 weeks (80–136). Group A received DOR plus abacavir/lamivudine (ABV/3TC) (*n* = 109) or tenofovir/emtricitabine (TFV/3TC) (*n* = 45). At week 48, the effectiveness of DOR plus ABV/3TC by ITT was 90.8% (CI_95_, 88.0–93.6), better than with TFV/FTC [73.3% (66.7–79.9); *P* = 0.003]. Only one virologic failure was observed. Mild adverse effects were the cause of treatment discontinuation in 7.8%, followed by switching to a single-tablet regimen. In group B, the effectiveness by ITT was 92.9% (CI_95_, 88.0–97.8) at week 48. No adverse effects or virologic failure were registered in this group. DOR plus two NRTI or DT have long-term effectiveness and safety as a switching option for PWH, mostly virologically suppressed. The DOR plus ABV/3TC combination has shown even better effectiveness than TFV/FTC.

**IMPORTANCE:**

DOR-based regimens have shown long-term effectiveness and safety in PWH, mostly virologically suppressed. The combination of DOR plus ABV/3TC has shown even better safety and effectiveness than TFV/FTC. DOR plus two NRTI offers cost benefits compared to other regimens.

## INTRODUCTION

Doravirine (DOR) is the last non-nucleoside reverse transcriptase inhibitor (NNRTI) approved as initial therapy in combination with two nucleos(t)ide reverse transcriptase inhibitors (NRTI), as well as for those virologically suppressed on antiretroviral treatment (ART) without resistance mutations to the planned drugs (1). This new drug provides advantages over other NNRTI, such as less frequent side effects, a favorable resistance profile, and infrequent interactions with commonly used drugs. Furthermore, it appears to have a higher genetic barrier and a lower risk of resistance mutation development than other NNRTI (2).

These recommendations are based on cumulative data from three clinical trials (DRIVE-FORWARD, DRIVE-AHEAD, and DRIVE-SHIFT). In the first two trials, DOR-based therapy demonstrated non-inferior efficacy compared to efavirenz (EFV) or ritonavir-boosted darunavir (DRV/rtv) plus two NRTI in treatment-naïve people with HIV (PWH) (3, 4). The last one demonstrated non-inferiority of DOR plus two NRTIs compared to boosted protease inhibitors (bPIs), cobicistat-boosted elvitegravir, or NNRTI plus two NRTI in participants with virological suppression. Furthermore, switching to DOR improved the lipid profile compared to bPIs-based ART (5).

On the other hand, in treatment-experienced patients with previous virological failures, a bPI-based regimen is usually chosen in clinical practice. However, bPI has to be frequently withdrawn or avoided due to drug interactions or tolerability. Moreover, resistance mutations that compromise DOR activity are rare in treatment-naïve (6, 7) and ART-experienced patients, even among subjects who fail other NNRTI (8). Therefore, in these clinical settings, DOR becomes a valuable alternative.

Although long-term safety and efficacy have been demonstrated in both treatment-naïve and experienced PWH in pivotal clinical trials (3, 5), real-world data often derive from short series with limited follow-up (9, 10). Furthermore, there is currently limited experience with DOR in dual therapy. This study aims to evaluate the effectiveness of DOR-based regimens in a real-world cohort of PWH, mostly virologically suppressed.

## MATERIALS AND METHODS

### Setting and study design

This observational, ambispective study enrolled adults PWH who initiated a DOR-based regimen from September 2020 to February 2022. The cohort was divided into three groups for its analysis: group A received DOR plus two NRTI. Group B received dual therapy with DOR plus dolutegravir (DTG) (*n* = 25) or darunavir/cobicistat (*n* = 3). Additionally, five subjects received DOR plus two or three additional antiretroviral drugs that, given the small number of participants, its analysis was carried out integrated into the overall cohort. The study was conducted at Virgen del Rocío University Hospital, Seville, Spain, a reference center with a median of 2800 PWH in active follow-up and approximately 100 new cases annually.

The study was designed and conducted according to the principles of the Declaration of Helsinki and the Good Clinical Practice. It was approved by the Committee on the Ethics of Research on Medicinal Products of Seville (FIS-DOR-2021-01) and registered at ClinicalTrials.gov (NCT05140603). There was an exemption from obtaining written informed consent, as demographic and clinical data were obtained from comprehensive, routinely collected, standardized electronic medical records encompassing patients under care at our center. According to our country’s prevailing legislation, it ensured that the information processed did not contain personal data.

### Participants

Eligible participants were adults (≥18 years) PWH who switched to a DOR-based regimen due to clinical considerations from the date of its availability at our center (September 2020) to July 2021 (retrospective part) and from August 2021 to February 2022 (prospective part). Previous failures on NNRTI, such as NVP or EFV, were accepted if only minor resistances to DOR were observed in the drug resistance mutation tests. By contrast, the presence of major resistance mutations to the study drugs was an exclusion criteria as well as pregnancy, active opportunistic infections, and use of immunomodulators or chemotherapeutic agents.

### Follow-up, assessment, and endpoints

The primary endpoints were treatment effectiveness at week 48 by intention-to-treat analysis (ITT), where non-complete or missing data were considered treatment failure, and per-protocol analysis (OT), in which all participants whose treatment was interrupted for nonvirological reasons were excluded. Treatment changes were allowed within NRTI due to toxicity or bad tolerability. The secondary endpoint included treatment effectiveness at 96 weeks with the above-mentioned criteria. Virological failure (VF) was defined as two consecutive plasma HIV−RNA >50 copies/mL or a single determination >200 copies/mL in case of loss to follow-up. The arbitrary cut-off point of ≥200 copies/mL to define VF based in only one sample was selected due to possible measurement errors in HIV RNA assays close to their limit of quantification (11, 12).

Subject assessments were performed at baseline and every 3–6 months thereafter, including adherence (subject self-report and pharmacy records), adverse events (AEs), biochemical and hematological profiles, flow cytometric counts of CD4^+^ and CD8^+^ T cells, and plasma HIV-RNA levels (COBAS 6800; Roche Diagnostics). AEs and abnormal laboratory findings were evaluated according to a standardized toxicity grade scale (AIDS Clinical Trials Group) (13). Furthermore, data of previous genotype resistance mutation tests according to the Stanford University HIV Drug Resistance Database were collected (14).

### Statistical analyses

Descriptive statistics were used for demographic, epidemiological, and clinical data. Results were expressed as median and interquartile range (IQR) for quantitative variables, number of cases and percentages for categorical variables, and proportions with 95% confidence interval (CI). The two-sided Wilcoxon signed rank sum test was performed to compare changes in continuous variables after the switch. Time-to-event analyses were performed using Kaplan-Meier survival curves to analyze effectiveness. Statistical analyses were performed with IBM software (SPSS, version 25.0; SPSS, Chicago, IL). *P*-values < 0.05 were considered significant.

## RESULTS

### Baseline characteristics of the cohort

A total of 191 patients were enrolled in the study; however, four individuals were lost to follow-up right after prescription and were excluded from the analysis. Therefore, a final cohort of 187 patients with a median follow-up of 112 weeks were analyzed (80–136), 154 in group A and 28 in group B. The baseline demographic, clinical characteristics of the study population, and reasons for switching to a DOR-based regimen are summarized in Tables 1 and 2, respectively. In group A, the median time of HIV infection was 12 years (8–21), the median of previous antiretroviral regimens was 5 (3–7), and 7 of 154 participants had detectable viral loads (63–1,640 copies/mL) before switching to a DOR-based regimen. In group B, these values were 15 (10–27.6) and 12 (7–15), respectively; 6 of 28 subjects had detectable viral loads (70–193.000) before switching to a DOR-based regimen. Regarding the total cohort, 158 (84.5%) participants were male, the median CD4^+^ T cell count was 683 cells/µL (525-877), and 170 patients (91%) had an undetectable viral load before switching to a DOR-based regimen.

**TABLE 1:** Baseline demographic and clinical characteristics of the study population

	DOR + 2 NRTI (*n* = 154)	Dual therapy (*n* = 28)	Others (*n* = 5)
Age, years	49 (42–57)	52 (43–58)	54 (37–57)
Gender, male	131 (85.1)	22 (78.6)	5 (100)
HIV infection, years	12 (8–21)	15 (10–28)	6 (4–28)
Risk factor for HIV			
MSM	84 (54.5)	12 (42.9)	1 (20.0)
Heterosexual	30 (19.5)	3 (10.7)	
IDUs	31 (20.1)	11 (39.3)	1 (20.0)
Others/unknown	9 (5.8)	2 (7.0)	1 (20.0)
CD4^+^/µL nadir	237 (111–347)	218 (97–311)	95 (35–311)
CD4^+^ T cell count/μL	687 (562–884)	612 (422–8234)	567 (289–762)
%CD4^+^ T cells	36.1 (29.4–41.4)	33.2 (25.0–40.2)	17.2 (14.8–35.6)
CD4^+^/CD8^+^ ratio	0.97 (0.64–1.25)	0.82 (0.47–1.08)	0.30 (0.22–1.10)
HIV-RNA ≤50 copies/mL	147 (95.4)	22 (78.6)	1(20.0)
ART^*a*^ prior to DOR	5 (3–7)	12 (7–15)	8 (5–22)
Resistance mutation	*n* = 59	*n* = 20	*n* = 5
M184V/I	5 (8.4)	13 (65.0)	
K103N/R/Q	8 (13.6)	7 (35.0)	3 (60.0)
1138K/A/G	1 (1.7)	4 (20.0)	1 (20.0)
G190A	1 (1.7)	2 (10.0)	1 (20.0)
Combinations of resistance mutations			
One mutation	11 (84.6)	14 (58.3)	1 (20.0)
Two mutations	2 (15.4)	8 (33.3)	2 (40.0)
Three mutations	0 (0)	2 (8.3)	2 (40.0)

^*a*^ ART, antiretroviral treatments. Data expressed as number (%) or median (interquartile range).

**TABLE 2:** Reasons for switching to a DOR-based regimen

Reasons for switching to a DOR-based regimen	*n* (%)
ART cost optimization	83 (44.4)
Specialist´s decision	52 (27.8)
Adverse effects	27 (14.4)
Drug-drug interactions	10 (5.3)
Virological failure	8 (4.3)
Other reasons	7 (3.7)

One hundred and forty-two participants (75.9%) were on triple therapy, mostly with 2 NRTI plus rilpivirine (RPV), and 41 patients (21.9%) on dual therapy (Table S1).

Previous drug resistance tests were available for 116 participants (62%), of which 32 samples did not show mutations or did not amplify due to virological failures with low viral load. Regarding resistance mutations related to prescribed treatments, in group A, five participants with de M184V/I, which confers a high level of resistance to 3TC and FTC, and an additional subject with the K103N, Y181C, G190A mutations conferring low-level resistance to DOR. In group B, one participant had the K103N, Y181C, and G190A mutations conferring high-level resistance to DOR, and four additional subjects with the Y181I, K103N/Y181C, E138K/M230I, and A98G, E138K, M230A mutations that confer low-level resistance to DOR. Group C consists of heavily treatment-experienced subjects, but only two of them had mutations that confer low-level resistance (K103N, Y181C, G190A) or intermediate resistance to DOR (K103R, V179F, Y181C, H221Y). Mutations related to PI and those unrelated to the treatments administered are not mentioned. Table S2 shows all the available genotypic resistance test.

### Doravirine-based regimens

In the group A (*n* = 154), the backbones were abacavir (ABV) plus lamivudine (3TC) (*n* = 109; 70.7%), tenofovir disopropil fumarate (TDF) plus emtricitabine (FTC) (36; 23.4%), and tenofovir alafenamide (TAF) plus FTC (9; 5.8%). On the other hand, in the group B (*n* = 28), the most common drug given together with DOR was DTG (25; 78.1%). In group C, two or more drugs were prescribed together with DOR (Table 3).

**TABLE 3:** Backbone therapy^*a*^

	Backbone therapy		*n* (%)
Group A	NRTI	ABC, 3TC	109 (78.8)
		TDF, FTC	36 (23.4)
		TAF, FTC	9 (5.8)
Group B	Dual therapy	DTG	25 (75.8)
		DRVc	3 (0.9)
Group C	Others	DTG, 3TC	2 (0.6)
		DRVc, 3TC	1 (0.3)
		DRVc, MRV	1 (0.3)
		RAL, TDF, FTC	1 (0.3)

^*a*^ NRTI, nucleos(t)ide reverse transcriptase inhibitors; ABC, abacavir; 3TC, lamivudine; TDF, tenofovir disopropil fumarate; FTC, emtricitabine; TAF, tenofovir alafenamide; DTG, dolutegravir; DRVc, darunavir/cobicistat; MRV, maraviroc; RAL, raltegravir.

### Effectiveness and safety

The effectiveness of DOR-based treatments in the whole cohort was 85.6% (CI_95_, 83.1%–88.2%) and 76.6% (CI_95_ 73.5%–79.7%) at week 48 and 96, respectively, by ITT, while that of DOR plus 2 NRTI was 85.7% (CI_95_, 82.9%–88.2%) and 77.8% (74.4%–81.2%), respectively. In the group A, the effectiveness of DOR plus ABV/3TC, without data in the literature, was better at 48 and 96 weeks compared to tenofovir (TVF)/FTC [73.3% (CI_95_, 66.7%–79.9%) and 62.2% (CI_95,_ 59.0%–73.4%) vs 90.8% (CI_95,_ 88.0%–93.6%) and 83.2% (CI_95,_ 79.6%–86.8%), *P* = 0.003] by ITT. However, the only virological failure with DOR plus 2 NRTI was with ABV/3TC. The effectiveness of the dual therapy was 92.9% (CI_95_, 88.0%–97.8%) and 80.9% (CI_95,_ 73.2%–88.6%) at week 48 and 96, respectively. The different analyses by ITT and OT are shown in Table 4.

**TABLE 4:** Percentage of participants without treatment failure by intention-to-treat analysis (ITT) and on treatment (OT) at 48 and 96 weeks^*a*^

	Backbone therapy		*n* (%)	ITT		OT	
				48 weeks	96 weeks	48 weeks	96 weeks
Group A	NRTI	ABV, 3TC	109 (78.8)	85.7% (CI_95_, 82.9–88.2)	77.8% (74.4–81.2)	99.3 (98.6–100)	
	(*n* = 154)	TDF, FTC	36 (23.4)				
		TAF, FTC	9 (5.8)				
Group B	Dual therapy	DTG	25 (75.8)	92.9% (CI_95_, 88.0–97.8)	80.9% (73.2–88.6)	100%	91.7% (86.1–97.3)
	(*n* = 28)	DRVc	3 (0.9)				

^*a*^ NRTI, nucleos(t)ide reverse transcriptase inhibitors; ABC, abacavir; 3TC, lamivudine; TDF, tenofovir disoproxil fumarate; FTC, emtricitabine; TAF, tenofovir alafenamide; DTG, dolutegravir; DRVc, darunavir/cobicistat.

Throughout the first 48 weeks, a total of 27 (14.4%) participants discontinued treatment due to AE (*n* = 13), lost to follow-up or treatment dropout (*n* = 7), change to a single tablet regimen (*n* = 3), VF (*n* = 3), and death (*n* = 1). From week 49 to 96, these values were AE (*n* = 1), lost to follow-up or treatment dropout (*n* = 3), change to a single tablet regimen (*n* = 9), and VF (*n* = 3) (Table 5).

**TABLE 5:** Reasons for discontinuation of treatment^*a*^

Backbone therapy		Discontinuation due to	0–48 weeks	49–96 weeks
Group A	NRTI (*n* = 154)	Adverse events	12	1
		Lost to follow-up	5	2
		Treatment dropout	1	1
		Patient’ choice (STR)	2	4
		Medical decision (STR)	1	5
		Virologic failure	1	
Group B	Dual therapy (*n* = 28)	Treatment dropout	1	
		Death	1	
		Medical decision^*b*^		1
		Virologic failure		2
	Others (*n* = 5)	Adverse events	1	
		Virologic failure	2	

^*a*^ STR, change to single tablet regimen.

^*b*^ Unconfirmed low-level viremia.

The reported AEs were gastrointestinal symptoms (*n* = 7), dizziness and nervousness, sleep disorder, and lack of concentration (*n* = 3), fatigue (*n* = 2), low back pain and arthralgia (*n* = 1). One additional participant presented an elevation of aminotransferase levels (grade 2). All AEs were mild and limited after treatment discontinuation. The only death reported was not related to treatment (community-acquired pneumonia in a patient with amyloidosis and chronic kidney disease on haemodialysis).

After 48 weeks, the CD4^+^ T cell count remained stable from baseline [683 cell/µL (525–880) vs 700 (495–908)] as is expected in subjects with a long time on treatment (15); however, the CD4/CD8 ratio increased slightly from 0.93 (0.57–1.22) to 0.98 (0.66–1.36), *P* = 0.056. On the other hand, lipid profiles and glomerular filtration rate remained without significant changes.

## DISCUSSION

Based on favorable properties shown in randomized pivotal trials (3–5), the latest European AIDS Clinical Society recommends DOR in a triple-drug tenofovir-based regimen (TDF/FTC or TAF/FTC) as an option to start ART and, together with the US Department of Health and Human Services, as an option to manage adverse effects, drug-drug interactions or to eliminate food requirements in virologically suppressed subject without resistant mutations to drugs in the planned regimen (2, 11). However, the real-life experience with DOR plus 2 NRTI is limited to a few cohort studies with small sample size and a follow-up of 24 or 48 weeks in the best scenario and limited to the above-mentioned combination (9, 10, 16). The main reason for switching to this DOR-based regimen was AEs, particularly related to dyslipidemia or weight gain, as in the studies by Ciccullo et al. (17) and Mazzitelli et al. (9), which included 33 and 132 patients, respectively. However, in both studies, the follow-up was limited to 24 weeks in most participants.

Two additional adult studies have evaluated the response to DOR/TDF/3TC as a single table regimen (STR) in adults PWH at 48 weeks. The first one enrolled a heterogeneous population, most virologically suppressed, in which 71 subjects received the above-mentioned combination. Other regimens were DOR plus ABC/3TC (*n* = 3), DOR plus DTG (*n* = 2), and DOR plus bictegravir/TAF/FTC (*n* = 1). Although the results are not disaggregated, the overall effectiveness by ITT was 83%, with an estimated CI_95_ of 74.6–91.4 (10). The last study enrolled 62 subjects receiving DOR/TDF/3TC, showing an effectiveness of 93.5% (estimated CI_95_, 87.4–99.6) at week 48 by ITT (16).

By contrast, our participants in group A (DOR plus 2 NRTI) received two pills as DOR plus TDF/3TC, as single table regimen are not available in Spain, which has probably adversely affected our results. One-third of the treatment failures in this group were due to switching to an STR. Another significant difference was that most participants (78.8%) received ABV/3TC rather than TDF/3TC, a combination for which there is no experience and guidelines are less certain about its efficacy (11). Despite this and the fact that our is a real-life study without the careful selection of subjects for their inclusion in a clinical trial, our results do not differ significantly for the DRIVE-SHIFT trial and the last two mentioned studies at week 48 (10, 16). Specifically, the effectiveness by ITT of the DOR plus ABV/3TC in our study was 90.8% compared to 90.4% for single-tablet DOR/TDF/FTC in the DRIVE-SHIFT and better than with TFV/FTC, with only one virological failure in a subject without previous resistance mutations. We cannot compare the effectiveness of our results at 96 weeks as there are no “real-life” studies with such an extended follow-up.

A not negligible reason for switching to a DOR-based regimen in the ABV/3TC group was ART cost optimization, which represented 44.4% of our population. At our center, a regimen of DOR plus ABV/3TC or TDF/FTC currently costs 166.6 to 176.5 € monthly, respectively, compared to other regimens, as DTG plus 3TC (420.4 € monthly) or bictegravir//TAF/FTC (480.4 € monthly).

Furthermore, primary drug resistance to DOR is very infrequent in Europe (18, 19). The genetic barrier of DOR appears to be higher compared to other drugs in the NNRTI class. Consequently, its prescription is safe in subjects without a history of VF on NNRTI. Nevertheless, caution should be exercised when multiple drug-resistance mutations have been selected within the NNRTI class (20). It should be noted that we did not observe virologic failures among the five participants with the M184V in the previous genotypic resistance tests.

On the other hand, the recommendation to use DOR plus DTG once daily is not included in any practice guideline since there is no evidence-based in clinical trials in contrast to the DTG plus RPV combination. However, compared with other NNRTI, DOR seems to have a better tolerability profile, fewer interactions with food or drugs, and a higher genetic barrier. Therefore, it is reasonable to choose this personalized regimen, especially in patients with comorbidities and polypharmacy, avoiding the use of bPI. Our data complement those of previous publications with 18 and 43 subjects with a follow-up of 12 and 28 weeks, respectively (21, 22), and two case series which included 21 and 85 participants with a median follow-up of more than 1 year (23, 24).

In participants on dual therapy, we found two virologic failures, none with previous resistant mutations to DOR or the companion drug. By contrast, there were no virological failures among those with previous resistance mutations to NNRTI.

In our cohort, 43 patients interrupted treatment with DOR, 14 of them because of AEs, most of them mild and reversible after drug discontinuation. It must be considered that, due to the characteristics of the study, many patients whose treatment was modified for pro-active switching manifested greater sensitivity to any AE of the new drug.

Among the main strengths of our study, it should be noted that, in addition to being the cohort with the largest sample size with a 2-year follow-up in real life, it is the only one that evaluates the efficacy of alternative combinations such as DOR plus ABV/3TC. This combination has shown good tolerance and long-term effectiveness, with advantages over TDF/FTC, such as less renal and bone toxicity. Furthermore, our cohort is representative of the HIV population currently treated in Central/Western Europe and Spain. Moreover, this study includes 25 patients on DTG/DOR dual therapy, most of them extensively treated, where the combination with DOR showed an efficacy of 100% in the OT analysis and a percentage of 92.9% in the ITT analysis at week 48.

Our study has some limitations: it was an observational, single-center one without a comparator arm. Moreover, since it was carried out during the COVID-19 pandemic, it has not been possible to report parameters such as weight changes, which would have been interesting to analyze. Instead, it is the most extensive reported series of DOR plus ABV/3TC, which was not included in clinical trials.

In conclusion, in our cohort, DOR-based regimens have shown long-term effectiveness and safety as an ART option for PWH, mostly virologically suppressed. The combination of DOR plus ABV/3TC, not tested as a switch strategy in clinical trials, has shown even better safety and effectiveness than TFV/FTC. Additionally, DOR plus two NRTI offers cost benefits compared to other regimens.

## SUPPLEMENTAL MATERIAL

Supplemental tables spectrum.00654-24-s0001.docx Tables S1 and S2.

ASM does not own the copyrights to Supplemental Material that may be linked to, or accessed through, an article. The authors have granted ASM a non-exclusive, world-wide license to publish the Supplemental Material files. Please contact the corresponding author directly for reuse.
