# Supplementary material for: Long-term effectiveness, safety, and tolerability of doravirine in antiretroviral-experienced people with HIV in real life
Source: Microbiol Spectr. 2024 Jun 25;12(8):e00654-24. doi: 10.1128/spectrum.00654-24 (PMC11302240; doi:10.1128/spectrum.00654-24)
Supplement: Supplemental tables — Tables S1 and S2. [file spectrum.00654-24-s0001.docx]

| ARV drugs previous to Doravirine | | | n (%) |
| --- | --- | --- | --- |
| Quadruple therapy | 2 NRTI + 1 PI + 1 InSTI | FTC, TAF, DRVc, EVGc | 1 (0.5) |
| Triple therapy | 2 NRTI + 1 NNRTI | 3TC, ABV, EFV | 2 (1.1) |
|  |  | 3TC, ABV, RPV | 83 (44.4) |
|  |  | FTC, TAF, EVF | 3 (1.6) |
|  |  | FTC, TAF, RPV | 13 (6.9) |
|  |  | FTC, TDF, RPV | 13 (6.9) |
|  | 2 NRTI + 1 PI | FTC, TAF, DRVc | 5 (2.7) |
|  | 2 NRTI + 1 InSTI | 3TC, ABV, RAL | 5 (2.7) |
|  |  | FTC, TAF, RAL | 1 (0.5) |
|  |  | FTC, TAF, BIC | 15 (8.0) |
|  | 1 NRTI + 1 PI + 1 InSTI | 3TC, DRVc, DTG | 2 (1.1) |
| Dual therapy | 1 NRTI + 1 InSTI | 3TC, DTG | 14 (7.5) |
|  | 1 NRTI + 1 PI | 3TC, DRVc | 9 (4.8) |
|  | 1 PI + 1 NNRTI | DRVc, RPV | 1 (0.5) |
|  | 1 PI + 1 InSTI | DRVc, DTG | 8 (4.3) |
|  |  | DRVc, RTG | 1 (0.5) |
|  | 1 NNRTI + 1 InSTI | RPV, DTG | 8 (4.3) |
| Monotherapy | 1 PI | DRVc | 3 (1.6) |

**Supplementary table 1**. Antiretroviral regimens before switching to a DOR-based regimen. NRTI: nucleos(t)ide reverse transcriptase inhibitors. NNRTI: non-nucleoside reverse transcriptase inhibitor. PI: proteasa inhibitor. InSTI: Integrase strand transfer inhibitor. FTC, emtricitabine. TAF, tenofovir alafenamide. DRVc, darunavir/cobicistat. EVGc, elvitegravir/cobicistat. 3TC, lamivudine. EFV, efavirenz. RPV, rilpivirine. RAL, raltegravir. BIC, bictegravir. DTG, dolutegravir.

| DOR + 2 NRTI  (n= 154) | n | Dual therapy  (n= 28) | n | Other combinations  (n= 5) | n |
| --- | --- | --- | --- | --- | --- |
| Resistance mutations |  | **Resistance mutations** |  |  |  |
| NRTi |  | **NNRTI** |  | **NRTI** |  |
| M184V/I | 1 | K103N | 1 | M41L, D67N, L210W, T215Y, K219R | 1 |
| M41L, M184V, L210W, T215Y | 1 | K103N, Y181C | 1 | **NNRTI** |  |
| M41L, E44D, D67N, T69D, M184V, L210W, T215Y | 1 | K103N, Y181C, G190A, F227L | 1 | K103R, V179F, Y181C, H221Y | 1 |
| K70N, T215A | 1 | E138K, M230I | 1 | K101P, K103N | 1 |
| M41L, M184V, T215Y | 1 | K103Q, Y181C | 1 | V179D, Y181C | 1 |
| D67N, M184V, T215C | 1 | Y181I | 1 | K103N, Y181C, G190A | 1 |
| NNRTI |  | K103N, E138A, G190A | 1 | E138A | 1 |
| K103N/R | 6 | A98G, E138K, M230A | 1 | **InSTI** |  |
| V106I | 1 | **InSTI** |  | E138K, G140S, Q148H | 1 |
| V90I, V106I | 1 | Q148K | 1 |  |  |
| K103R, E138G | 1 |  |  |  |  |
| V179D, Y181C | 1 |  |  |  |  |
| K103N, Y181C, G190A | 1 |  |  |  |  |

**Supplementary Table 2**. Resistance mutations before switching to a doravirine (DOR)-based regimen. NRTI, nucleos(t)ide reverse transcriptase inhibitors. Dual therapy, DOR plus dolutegravir (n= 25) or darunavir/cobicistat (n=3).
